# VPS-34 Inhibition Ameliorates Postural Aging by Regulating GABAergic Motor Neuron Activity in C. elegans

**DOI:** 10.1093/geroni/igaf122.2548

**Published:** 2025-12-31

**Authors:** Xuhui Chen, Le Zhang, Shangbang Gao, Cuntai Zhang

**Affiliations:** Huazhong University of Science and Technology, Wuhan, Hubei, China (People’s Republic); Huazhong University of Science and Technology, Wuhan, Hubei, China (People’s Republic); Huazhong University of Science and Technology, Wuhan, Hubei, China (People’s Republic); Huazhong University of Science and Technology, Wuhan, Hubei, China (People’s Republic)

## Abstract

Motor aging encompasses various functional and postural phenotypes, including reduced movement speed and increased body stiffness and curvature. These phenotypes reflect physiological aging in multiple organs, such as bones (calcium loss, reduced collagen content and osteoporosis) and muscles (atrophy, decreased elasticity, reduced endurance and flexibility). However, the neurobiological mechanisms regulating motor aging, particularly the specific molecular pathways and potential interventions, are not well understood. In this study, we used Caenorhabditis elegans to model motor aging. Aging worms exhibited reduced movement speed and increased body curvature, similar to human aging phenotypes. Investigations into synaptic morphology and neurotransmission function at C. elegans neuromuscular junction revealed that decreased excitatory motor neuron activity contributed to the reduction in movement speed. Notably, an imbalance between excitatory and inhibitory signaling resulted in the increased body curvature. Mutation of the phosphoinositide 3-kinase (PI3K) VPS-34, a key regulator of endosomal trafficking, alleviated the curvature defects associated with aging. This alleviation was linked to enhanced inhibitory GABAergic motor neuron activity, suggesting a protective role of VPS-34 inhibition in postural aging. Our findings demonstrate that different motor aging phenotypes are regulated by distinct neural mechanisms and VPS-34 regulates postural aging by modulating inhibitory motor neuron activity. These results thus offer new insights into VPS-34 inhibition as a potential neuro-intervention strategy for mitigating motor aging.

